# 157. A Multicenter, Mixed-Method Evaluation of Delayed Hospital Discharge in Patients with Invasive Candidiasis Receiving Echinocandins

**DOI:** 10.1093/ofid/ofab466.359

**Published:** 2021-12-04

**Authors:** Jinhee Jo, Joshua Hendrickson, Masaad Almutairi, Faris S Alnezary, Nicholas Beyda, Anne J Gonzales-Luna, Truc T Tran, Debora Simmons, Kevin W Garey

**Affiliations:** 1 University of Houston, Houston, Texas; 2 UPMC, Pittsburgh, Pennsylvania; 3 University of Houston College of Pharmacy, Houston, Texas; 4 Center for Antimicrobial Resistance and Microbial Genomics, UTHealth, Houston, TX, Houston, TX; 5 University of Texas Health Science Center, Houston, Texas

## Abstract

**Background:**

Patients with systemic candidiasis often receive prolonged echinocandin therapy in the inpatient or outpatient setting. Rezafungin is a novel echinocandin currently in clinical trials characterized by once-weekly dosing interval. In order to understand the potential benefit of rezafungin to facilitate earlier hospital discharge, the purpose of this project was to better understand barriers to discharge in patients with proven or suspected invasive candidiasis.

**Methods:**

Electronic health records from two large health systems (20+ hospitals) were reviewed to identify patients given an echinocandin. Patients given an echinocandin until hospital discharge were evaluated for outpatient use as well as barriers that prevented earlier discharge. Identified barriers were developed into a quantitative framework and a qualitative interview guide. Using a constant comparative method, the framework for hospital discharge barriers was constructed using a series of open-ended questions and axial coding to identify discharge barrier themes. Results were integrated to produce a mixed-method model.

**Results:**

A total of 1,665 echinocandin courses were evaluated. Five hundred and thirty-four patients (32%) received echinocandin therapy until at least the day of hospital discharge of which 328 of 534 (61%) patients were either discharged to home or transferred to another facility. Significant predictors for outpatient echinocandin use were osteomyelitis (OR 4.07, 95% CI: 1.06-15.66; p=0.041) and other deep-seated infection (OR 4.44; 95% CI: 1.65-11.96; p=0.003). Stewardship analysis identified the majority of patients (54%) had the possibility for at least one day earlier discharge (potential earlier discharge:1.65±1.16 days). The quantitative model identified major barriers to be transition of care-, other medical care-, and infectious diseases-related. The qualitative model largely agreed with the quantitative model with additional psychosocial and health care access variables identified.

**Conclusion:**

Using a mixed method approach, barriers to hospital discharge and potential use of new antifungal therapies were identified. These data could be used to assist transitions of care in patients with invasive candidiasis.

**Disclosures:**

**Truc T. Tran, PharmD**, **Merck** (Grant/Research Support) **Kevin W. Garey, Pharm.D., M.S., FASHP**, **Summit Therapeutics** (Research Grant or Support)

